# Improved Outcomes in Operative Management of Concomitant Distal Radius and Scaphoid Fractures

**DOI:** 10.1177/15589447231163943

**Published:** 2023-04-12

**Authors:** Luke Verlinsky, Clinton Ulmer, Alec Rose, Christina Brady, Ryan Rose

**Affiliations:** 1 Department of Orthopaedics, UT Health San Antonio, TX, USA; 2 Scripps Mercy Hospital, San Diego, CA, USA

**Keywords:** concomitant fractures, scaphoid, wrist, fracture/dislocation, distal radius, outcomes, research & health outcomes, treatment

## Abstract

**Background:**

This study aims to investigate the characteristics of concomitant distal radius and scaphoid fractures and determine outcome differences of operative and nonoperative management.

**Methods:**

A retrospective search of a level-1 trauma center’s database over a 15-year period (2007-2022) for concomitant distal radius and scaphoid fractures in adult patients was completed. In all, 31 cases were reviewed for mechanism of injury, method of fracture management, distal radius fracture AO Foundation/Orthopaedic Trauma Association classification, scaphoid fracture classification, time to radiographic scaphoid union, time to motion, and other demographics. A multivariate statistical analysis was completed comparing outcomes in operative versus conservative management of the scaphoid fracture in these patients. Outcomes were defined as time to radiographic union and time to motion.

**Results:**

In all, 22 cases of operative fixation of the scaphoid and 9 cases of nonoperative management of the scaphoid were reviewed. One case of nonunion was identified in the operative group. Operative management of scaphoid fractures resulted in a statistically significant reduction in time to motion (2-week reduction) and time to radiographic union (8-week reduction).

**Conclusions:**

This study demonstrates that operative management of scaphoid fractures in the setting of a concomitant distal radius fracture reduces the time to radiographic union and time to clinical motion. This suggests that operative management is ideal in patients who are good candidates for surgery and desire earlier return of motion. However, conservative management should be considered, as nonoperative care showed no statistical difference regarding union rates of scaphoid or distal radius fractures.

## Introduction

Distal radius fractures are an exceedingly common injury pattern. Fractures involving the distal radius account for one-sixth of all fractures, with over 640,000 cases reported during 2001 in the United States alone.^1^ Extensive research exists regarding the epidemiology, classification, management, and complications of isolated distal radius fractures.^2^ However, discussion of the concomitant distal radius and scaphoid fractures is rare, with only 163 total cases published in the literature.^3-18^ Of these, only 70 cases have been reported since the introduction of volar locking plates in 2002.^3-6^,^18^ The incidence of an ipsilateral scaphoid fracture has been reported up to 4% to 4.7% of all distal radius fractures.^5,15^ An incidence of 4% of concomitant distal radius and scaphoid fractures with only 163 total reported cases suggests that this injury pattern is grossly underappreciated in our current literature.

This fracture pattern presents unique peri- and postoperative management difficulties, as scaphoid and distal radius fractures reductions occur in opposite vectors. Distal radius fracture reduction requires longitudinal traction and extension to flexion movement, while the scaphoid requires compression. Reduction maneuvers for the distal radius can theoretically displace the scaphoid in the process. The competing forces at work in management pose challenges during the operative or nonoperative management of these fractures. While there is an abundance of literature regarding the postoperative care of isolated distal radius or scaphoid fractures,^19,20^ the ideal timeline of recovery and method of immobilization in concomitant injuries is unclear.

Given the deficiency of the current literature and the unique challenges this fracture pattern presents, the goal of this study is to further the knowledge of clinical and radiographic outcomes of concomitant distal radius and scaphoid fractures. To our knowledge, this study has the largest n-value of combined distal radius and scaphoid fractures to date. We hypothesize that this injury pattern can be managed effectively based on current AO guidelines. Additionally, that incidence of concomitant distal radius and scaphoid fractures matches the literature at 4% to 4.7%. We also hypothesize that nonoperative management of the combined injury results in longer times of radiographic union and immobilization.

## Methods

After institutional review board approval, the trauma database of a level-1 trauma center was queried for all patients treated for a distal radius fracture over a 15-year period (2007-2022). All adult trauma activations between 2007 and 2022 were reviewed for a diagnosis of distal radius and scaphoid fracture. Of all distal radius fractures, 91 had concomitant scaphoid fractures. The records of the 91 patients with combined injuries were reviewed to ensure injury pattern, patient characteristics, and documentation met inclusion criteria. Criteria required patients to be over the age of 18 and have sustained ipsilateral distal radius and scaphoid fractures in the same injury event. Patients without adequate clinical and radiographic follow-up to the point of bony union were excluded. Two patients were excluded from the analysis as the wrist was immobilized for an extended period utilizing a dorsal bridge plate in 1 case and an external fixator in the other due to severe pan-carpal injuries. Chart review included age, gender, mechanism of injury, method of fracture management, distal radius fracture AO Foundation/Orthopaedic Trauma Association (AO/OTA) classification, scaphoid anatomic classification, time to radiographic scaphoid union, and time to motion.

All cases of combined distal radius and scaphoid fractures were reviewed radiographically by 2 orthopedic trained hand surgeons. In addition to x-ray, computed tomography scans were reviewed for confirmation when available. Scaphoid fractures were classified as proximal pole, waist, or distal pole fractures. Outcomes were defined as time to union and time to motion, as determined from subsequent radiographs and follow-up documentation. Union was determined radiographically when >50% bridging trabeculae could be visualized at the fracture site. Computed tomography scans were utilized when available. Chi-square test was utilized to compare categorical variables. Student *t*-test was utilized to compare age and time to motion; the Wilcoxon rank sum test was utilized to compare time to union and follow-up length between operative and nonoperative groups. A multivariate regression analysis was utilized to adjust for age as a confounding variable.

## Results

Out of all cases of distal radius fractures in adults over a 15-year period, 91 cases (4.5%) of concomitant scaphoid fractures were identified. Thirty-one cases with adequate clinical and radiographic follow-up were included for analysis. The majority of concomitant distal radius and scaphoid fractures were associated with high-energy trauma (87.2%) and occurred more commonly in males (80.7%). In addition, most cases were associated with AO/OTA type C distal radius fractures (71%). In 9 cases, the scaphoid fracture was managed conservatively. In 22 cases, the scaphoid fracture was managed operatively with either percutaneous screw fixation or open reduction and internal fixation. Patients were followed clinically for an average of 32 weeks postinjury. There were no significant differences between groups with regard to gender, open fracture, distal radius fracture AO/OTA classification, scaphoid fracture characteristics, or follow-up length. One case of nonunion was identified in the operative group. However, in all other cases, time to union was significantly shorter in the operative group (6.0 weeks ± 2.0) than the nonoperative group (13.7 weeks ± 11.7) (*P* = .0352). In addition, time to motion was significantly shorter for patients in the operative group (5.7 weeks ± 2.0) than the nonoperative group (7.4 weeks ± 2.3) (*P* = .0409). There was a statistically significant difference in age between the operative (30.3 years ± 9.2) and nonoperative (45.4 years ± 14.6) groups (*P* = .0015). When controlling for age utilizing multivariate regression, the difference in time to union and time to motion remained statistically significant (*P* = .0424, *P* = .0446) (Table 1).

**Table 1.** Patient Characteristics and Scaphoid Management.

	Overall, n = 31	Non-op scaphoid management, n = 9	Operative scaphoid management, n = 22	*P* value
Mean age ± SD, yrs	34.7 ± 12.8	45.4 ± 14.6	30.3 ± 9.2	.0015
Gender				
Male	25 (80.7%)	7 (77.8%)	18 (81.8%)	NS
Female	6 (19.3%)	2 (22.2%)	4 (18.2%)	
Mechanism of injury				
Fall from >3 m	14 (45.2%)	4 (44.4%)	10 (45.4%)	
MVC	4 (12.9%)	1 (11.1%)	3 (13.6%)	
MCC	7 (22.6%)	2 (22.2%)	5 (22.7%)	
MV vs pedestrian	2 (6.5%)	2 (22.2%)	0	
Fall from <3 m	4 (12.9%)	0	4 (18.2%)	
Open distal radius fracture	6 (19.4%)	1 (11.1%)	5 (22.7%)	NS
Distal radius fracture AO/OTA classification				
A	2 (6.5%)	1 (11.1%)	1 (4.6%)	NS
B	7 (22.6%)	1 (11.1%)	6 (27.3%)	
C	22 (71%)	7 (77.8%)	15 (68.2%)	
Scaphoid fracture location				
Distal pole	4 (12.9%)	3 (33.3%)	1 (4.6%)	NS
Proximal pole	2 (6.5%)	0	2 (9.1%)	
Waist	25 (80.7%)	6 (66.7%)	19 (86.4%)	
Distal radius intervention				
Closed reduction	7 (22.6%)	5 (55.6%)	2 (9.1%)	
ORIF	21 (67.7%)	3 (33.3%)	18 (81.8%)	
Ex-Fix/ORIF	1 (3.2%)	0	1 (4.6%)	
CRPP	2 (6.5%)	1 (11.1%)	1 (4.6%)	
**Time to union** ^a^ **(mean time ± SD, wks)**	**8.3 ± 7.3**	**13.7 ± 11.7**	**6.0 ± 2.0**	.**0352/.0424**^b^
**Time to motion (mean time ± SD, wks)**	**6.2 ± 2.2**	**7.4 ± 2.3**	**5.7 ± 2.0**	.**0409/.0446**^b^
Follow-up (mean time ± SD, wks)	32.1 ± 44.4	45.3 ± 56.8	26.7 ± 38.5	NS

*Note.* Nonoperative and Operative columns describe scaphoid fracture management. SD = standard deviation; AO/OTA = AO Foundation/Orthopaedic Trauma Association; MVC = motor vehicle collision; MCC = motorcycle collision; ORIF = open reduction internal fixation; Ex-fix = external fixation; CRPP = closed reduction percutaneous pinning.

a One case of nonunion in operative group excluded from statistical analysis.

b *P*-value after multivariate regression controlling for age.

## Discussion

Distal radius fractures are exceedingly common, accounting for approximately 17% of all fractures.^1^ Based on previous literature, 4% to 4.7% of all patients with a distal radius fracture also have a scaphoid fracture.^5,15^ Extrapolating this incidence of concomitant scaphoid fractures to the 640,000 recorded distal radius fractures in the United Stated in 2001 estimates that between 25,000 and 30,000 of those patients had concomitant scaphoid fractures. Despite this, there are very few cases described in the literature during the last 20 years. The lack of published outcomes handicaps the creation of evidence-based guidelines for management.

Current literature recommends operative management.^21^ Since the proliferation of volar locking plates for distal radius fractures in the early 2000s, there have been 5 publications presenting clinical data regarding concomitant fractures (Table 2).^3,5,6,17,18^ Slade et al^17^ discussed 7 patients that were treated in a stepwise approach of percutaneous scaphoid pinning, followed by open reduction and internal fixation of the distal radius and then screw placement in the scaphoid. Rutgers et al^6^ reported on 10 patients in which 9 were treated operatively. Those authors guided the treatment protocol based on the characteristic and the severity of the distal radius fracture. Komura et al^5^ detail 8 patients with 4 distal radius and 5 scaphoid fractures treated operatively. However, their publication was descriptive in nature with no outcomes reported. Gürbüz et al^18^ describe 22 combined injures which were all treated operatively. This series showed an approximately 80% decrease range of motion of the operative wrist at a mean follow-up of 25 months. Finally, Fowler and Fitzpatrick^3^ outlines 23 patients, 3 of whom were lost to follow-up, who were all treated with volar locking plates and scaphoid screw fixation. These authors report the only scaphoid nonunion among the above-mentioned series. This nonunion occurred in connection with an ipsilateral brachial plexopathy.

**Table 2.** Brief Characteristics of Prior Combined Distal Radius and Scaphoid Fracture Studies.

Author	Cases	Op vs Non-op	Radius treatment	Scaphoid treatment	Outcomes	Complications
Fowler and Fitzpatrick^3^	20	20/0	VLP: 12 dorsal plate: 1 VLP + dorsal plate:2 K-Wire:1 Ex-fix (EF):4	Screw: 20	-	Nonunion: 1 Carpal tunnel release: 5
Gürbüz et al^18^	22	22/0	Screw: 1 EF + K-wire: 3 K-wire: 4 VLP: 14	Screw: 20 K-wire: 2	Flex/Ext/RD/UD: 45/48/20/43 Grip/pinch: 31/8.5 kg	Ulnar nerve entrapment: 1
Komura et al^5^	8	8/0	Cast: 4 Screw: 1 EF + K-wire: 1 VLP:2	Screw: 3 Cast: 5	-	
Rutgers et al^6^	9	8/1	Cast: 1 Screw: 1 EF: 1 Dorsal plate: 5 VLP: 2	Screw: 9 Cast: 1	Flex/Ext/RD/UD: 57/71/12/31	Screw loosened: 1 Carpal tunnel syndrome: 3 Proximal pole avascular necrosis: 1
Slade et al^17^	7	7/0	VLP: 7	Screw: 7	Flex/Ext/RD/UD: 50/65/15/26 Grip: 40 kg	Extensor pollicis longus rupture: 1

*Note.* VLP = volar locking plate; K-wire = Kirschner wire; RD = radial deviation; UD = ulnar deviation.

Our institution found that of all distal radius fractures identified in the 15-year period, approximately 4.5% of the fractures had concomitant scaphoid fractures. This incidence is comparable to the reported incidence of other studies. In all, 80% of scaphoid fractures in this study occurred at the waist, which is consistent with the current literature that suggests that waist fractures occur between 66% and 82% of the time.^3,15^

The requirement for high-energy forces needed during simultaneous fracture of the scaphoid and radius has been previously discussed.^5,14^ The injury forces seen in our data followed a similar trend. The 87.2% of the cases reviewed in this study resulted from high-energy mechanisms, and 71% of the distal radius fractures were AO/OTA type C fractures.

Management options include closed reduction and immobilization, open reduction and internal fixation, closed reduction and percutaneous pinning, or bridging constructs (external fixator or dorsal bridge plate).^6^ Several authors have shown satisfactory outcomes from nonoperative treatment of a distal radius fracture and scaphoid fracture.^7,9,11,15^ However, most of the patients in those studies had extra-articular fractures of the radius and no displacement of the scaphoid. Other authors that observed intraarticular fractures of the distal radius and displaced scaphoid fracture used operative approaches for treatment.^10,14,17^ Most patients in our study presented in a trauma setting, largely from a high-energy mechanism of injury. The high-energy trauma frequently resulted in more frequent intraarticular fractures or displacement of the scaphoid. In this study, operative management of the scaphoid fracture was utilized in 66% of the cases. We agree with Rutgers et al that typically, the characteristic and severity of distal radius fracture should dictate management. Operative management of the scaphoid fractures were performed utilizing either an antegrade or retrograde headless compression screws placed percutaneously or with an open surgical approach.

This study demonstrates that operative management of scaphoid fractures in the setting of a concomitant distal radius fracture reduces the time to radiographic union by approximately 8 weeks, and the time to motion clinically by approximately 2 weeks. This suggests that operative management is ideal in patients who are good candidates for surgery and desire earlier return of motion. Early surgical management can potentially hasten the return to work for many of the patients, reduce atrophy, and improve the ability to perform activities of daily living. Especially in the setting of distal radius fracture operative fixation, additional scaphoid surgery does not pose a much greater risk to the patient, as an antegrade headless compression screw can be placed in the scaphoid either percutaneously or with minimal additional incision.

Despite the benefits of operative management, nonoperative care must be considered. Of the 80 cases presented in the literature over the past 20 years, only 1 patient received no surgical intervention. In contrast, 9 of 31 patients were treated nonoperatively in this study, and all 9 cases achieved bony union, although 2 resulted in delayed union. In this study, 1 case of operative scaphoid fixation led to asymptomatic nonunion. However, this patient sustained a distal pole fracture which was treated with an antegrade compression screw with insufficient purchase on the distal fragment. Despite a singular case of nonunion, nonoperative care showed no statistical difference regarding union rates of scaphoid fractures. Scaphoid nonunion in combined injuries is quite rare. Of the last 107 reported cases in the literature, only 1 scaphoid nonunion occurred with a concomitant ipsilateral brachialplexopathy.^3^

This study has several limitations. The facility is a large level-1 trauma center that sees a high volume of high-energy trauma, resulting in a higher rate of operative management and a higher proportion of more severe or comminuted injuries than in the community. Low-energy trauma patients with distal radius fractures were less likely to be included in this data set. Additionally, the trauma population has generally poor follow-up potential. Only 33 of 91 patients identified had adequate follow-up documented, and 2 patients were excluded due to significant pan-carpal injuries requiring prolonged immobilization. Of the 31 patients included in analysis, follow-up was maintained for an average of 32 weeks, which was sufficient to determine union and motion outcomes. In further studies, the use of objective hand outcomes and patient-reported outcomes would be useful as an additional piece of data in evaluating outcomes in different treatment groups. Additionally, a worthwhile endeavor would be to investigate outcome differences between operative and nonoperative management of scaphoid fractures in the setting of operatively managed distal radius fractures. In this study, there was no sufficient power to perform this analysis, as there were only 4 cases of nonoperatively managed scaphoids with operative distal radius fractures. Nevertheless, this study greatly increases the total number of reported cases of concomitant scaphoid and distal radius fractures and sheds light on the improved outcomes in operative management methods.

The results from this study can further guide surgeons’ management of concomitant distal radius and scaphoid fractures. This series provides additional information about operative and nonoperative care of concomitant distal radius and scaphoid fractures. Informed and shared decision-making remains paramount when a provider and patient are developing a treatment plan.
